# Supplementary figures and images for: Evaluation of two automated low-cost RNA extraction protocols for SARS-CoV-2 detection
Source: PLoS One. 2021 Feb 16;16(2):e0246302. doi: 10.1371/journal.pone.0246302 (PMC7886139; doi:10.1371/journal.pone.0246302)

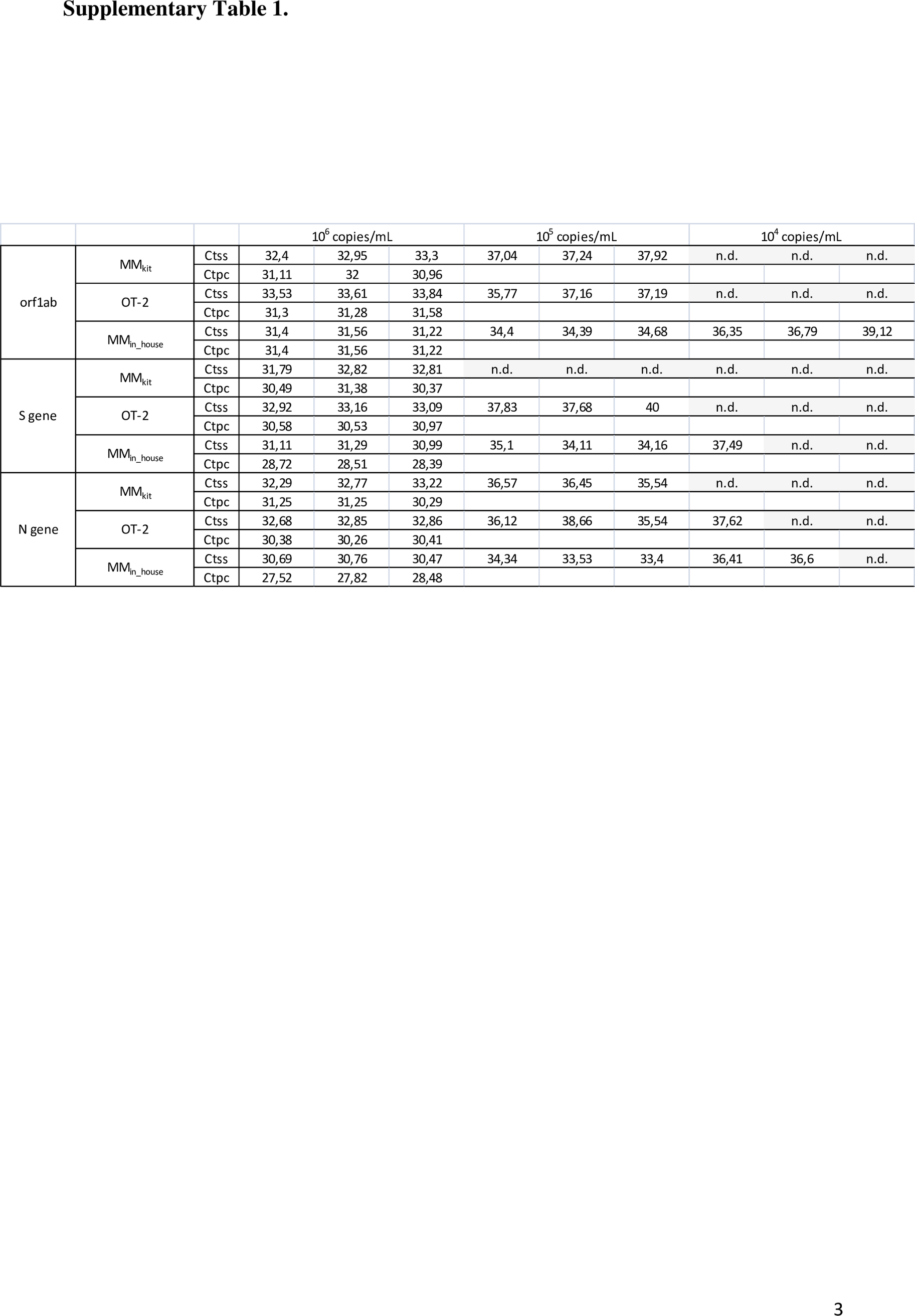

Supplement: S1 Table — (n.d. not detected). (TIF) [file pone.0246302.s001.tif]

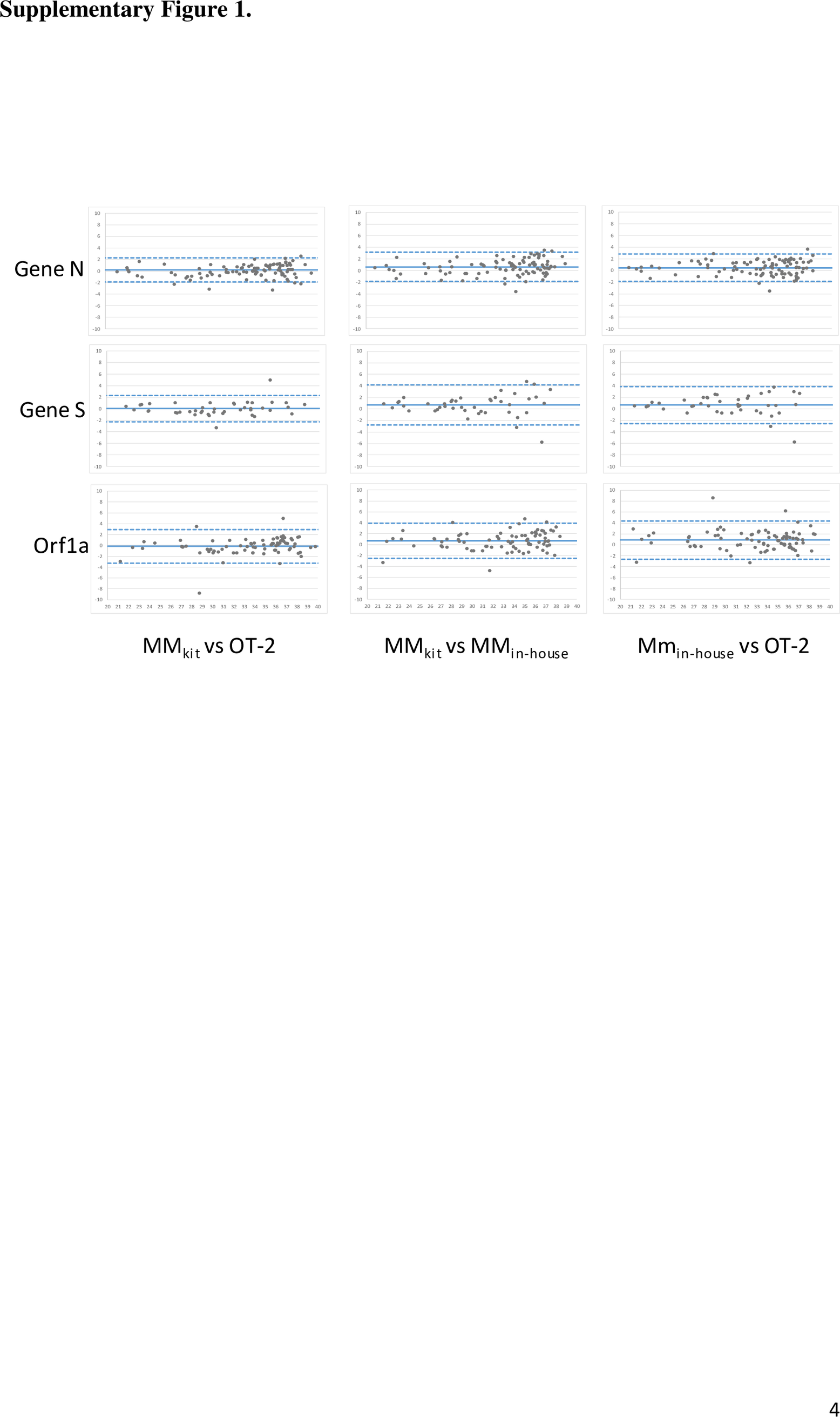

Supplement: S1 Fig — The same set of samples was extracted with the three methods and analyzed with a commercial PCR targeting three marker genes. The horizontal axes show the average Cts, and the vertical axes show the difference between Cts for each sample with the two methods indicated. The horizontal continuous line marks the average difference, and the discontinuous lines indicate the 95% limits of agreement (average difference ± 1.96 standard deviation of the difference). (TIF) [file pone.0246302.s002.tif]
